# Hospital efficiency measurement in the west of Iran: data envelopment analysis and econometric approach

**DOI:** 10.1186/s12962-022-00341-8

**Published:** 2022-02-09

**Authors:** Mohamad Yousefi Nayer, Ali Akbar Fazaeli, Yadollah Hamidi

**Affiliations:** 1 Department of Health Management and Economics, School of Public Health, Hamadan University of Medical Sciences, Hamadan, Iran; 2 Department of Health Management and Economics, School of Public Health, Tehran University of Medical Sciences, Tehran, Iran; 3 Department of Health Management and Economics, School of Public Health, Hamadan University of Medical Sciences, Hamadan, Iran

**Keywords:** Data envelopment analysis, Hospital, Efficiency, Tobit model

## Abstract

**Objective:**

Measuring hospital efficiency is one of the way how to use resources.The optimal hospital performance is the goals of healthcare policymakers. This study aimed to the current study was conducted to evaluate the efficiency the current study was conducted to evaluate the efficiency and assess the association between hospital size and hospital area population with technical efficiency in public hospitals.

**Methods:**

In this descriptive-analytical study, the statistical population consisted of 15 public hospitals in the west of Iran. First, the data envelopment analysis (DEA) method was used to evaluate technical efficiency. inputs included staff and beds, and outputs consisted of the number of surgeries, the number of patients, and the average length of stay. Then, according to the public ownership of all hospitals, their educational and therapeutic activities, as well as their size and population were considered as the environmental factor affecting efficiency. Thus, regression was applied to measure their effects on efficiency.

**Results:**

The average technical efficiency of the studied hospitals, the average management efficiency, and the average efficiency of the scale were 0.935, 0.961, and 0.987, respectively. Out of the total evaluated hospitals, six and nine hospitals had an efficiency of less than one and one, respectively. Moreover, the size of the hospital and the population as the environment variable were significant in the Tobit model. Our regression demonstrated that although the size of the hospital is positively associated with its technical efficiency, the hospital population negatively affects hospital efficiency.

**Conclusion:**

According to the size and area population of the hospitals, they decrease their inputs to maximize their efficacy by optimizing their surplus amounts. Tobit regression analysis concludes that hospital size and population covered by the hospital significant effect on hospitals' efficiency.

## Introduction

Health has a significant impact on the infrastructure of different parts of society based on sustainable social, economic, political, and cultural development [1]. The promotion of health is a moral obligation, including the social and economic categories. Thus, every health treatment service planning should be part of the pervasive attitude of healthcare policy to make part of the integrated plan of sustainable development [2]. Most of the world’s countries have faced rising health treatment section expenses during the recent decade [3]. This problem has occurred due to the combination of factors associated with demand, including demographic and epidemiological changes, the developed technology, and the lack of available information for health treatment services’ customers and consumers related to factors affecting supply. Moreover, the previous research shows that at least some parts of the rising expenses result from the inefficient use of sources [4]. Similar to major organizations, hospitals play a critical role in providing health treatment services, resulting in the recovery facilitation of the physical and mental health of the patient in society in a specific way. It also leads to training specialists in the health treatment section, performing medical research, and improving the health of society. Therefore, this organization has a particular sensitivity and importance regarding economic infrastructures and its severe vulnerability against currency rate fluctuations and commodity markets, particularly in developing countries [5]. This institution is the most extensive and most costly operating unit of the health treatment system in Iran [6]. The portion of hospital expenses is estimated at least 40% of total health expenses in 2015. This statistic is out of the total expenses of health treatment based on the national health accounts of Iran [4].

Considering that the health change plan was implemented in May 2014 with particular effects on hospitals, the portion of health expenses has increased out of gross domestic incomes, and that of the hospitals has risen through this statistic more than ever [7]. In Iran, public hospitals are funded by the government. The rise in demand, the rapid growth of health expenses to incomes in developing countries, economic crises, and the government budget lack are many problems for hospitals. Therefore, hospitals sustain heavy pressure to control and decrease expenses. These conditions double the necessity of creating additional probable resources and using the possible resources with resource allocation patterns and increasing efficiency in this section [8].

There is a pressing need concerning the optimal use of scarce sources and improvement of efficiency for providing health treatment cares. Accordingly, some measures are taken into account to prevent or decrease the waste of resources allocated to the health treatment system, helping in providing services as better as possible, developing availability, and improving hospital service quality [9]. Therefore, the use of scientific and applied methods is necessary for assessing the function and activities of the hospital and the optimal use of physical and human resources. Thus, one can enjoy economic tools and analyses, which provide a logical and specific framework for analyzing vital health care subjects. Although the mere assessment of health treatment services seems unnecessary, due to their human and humanitarian nature, it is highly beneficial to use assessment in the measurement, efficiency, and optimal use of resources [10].

In the economic literature, efficiency is the minimum use of inputs for a certain level of output. In other words, it is the increasing of outputs with a certain level of inputs. Inputs are the same as manufacturing factors such as energy, initial materials, capital, and labour force in the manufacturing process of commodities and services for generating outputs (i.e., commodities or services in the organization). Efficiency is an appropriate criterion for measuring the acquirement of the best output by limited inputs. Further, it is a new approach to peoples’ work and life [11]. The ratio analysis can be used in most efficiency studies. This method has limitations due to its use in the measurement of efficiency between one input and one output and the association of the studied subject for creating a relation between one input and some outputs or some inputs and some outputs. It is noteworthy that more advanced methods should be used since a hospital is an organization that encounters some inputs and outputs [12]. Extensive studies have focused on assessing the hospital, including Mahfoozpour et al. [13], Kiadaliri et al. [14], Rezaei et al. [15, 16], Mosadeghrad et al. [17], Hatam et al. [18], Goudarzi et al. [19] in Iran as well as Mujasi [20], Cheng, [21], and Ali [22] in Uganda, China, and Ethiopia, respectively. Therefore, there is now a great interest in data envelopment analysis as a dynamic, capable, and progressive method for measuring efficiency and productivity. It is mainly applied in government units and the private sector, in which their valuable information is unavailable or unreliable.

Data envelopment analysis determines whether the considered decision-making units consider the efficiency line. It is non-parameter linear planning that estimates the frontier production function. The difference between this study and previous studies is Tobit regression, which can measure the effect of environmental factors on the efficiency level. The environment in this context is the factor that can affect firm efficiency although it is not part of the applied inputs. Furthermore, the assumption is that it is not under management control. Environment variables are ownership, the number of customers, and the firm position and size. Thus, if a dependent variable of a critical limit, Tobit regression or censored regression, will apply to review linear relationships [23]. Considering the above-mentioned explanations, the current research sought to evaluate the technical efficiency of the public hospitals of Hamedan County with data envelopment analysis and Tobit regression in 2018.

## Methods

### Data collection

The population of this descriptive study includes all public hospitals (N = 15) in the west of Iran. Due to ethical considerations, the name of the hospital is specified in alphabetical order in this study. Data collection tools for the theoretical framework of the research are scientific and library documentation. Given that data are collected from hospitals by standard tables of the Ministry of Health and Medical Education, there is no need to determine validity and reliability. Some reports were used to collect data consisting of data in 2018 such as general particularities of the hospital, the number of fixed and active beds, all patients (outpatients and inpatients), the number of staffs (i.e., physicians, nurses, and other staff), the number of surgeries (i.e., emergency, outpatient, and standard), and the length of stay.

### Statistical analyses

Data envelopment analysis was used to analyze data and assess the efficiency level by Deap 2.1, assuming variables return to scale, which was of input-based type. The mathematical relation of data envelopment analysis is as follows:

$$\begin{gathered} Max\, Z_{0} = \mathop \sum \limits_{r = 1}^{s} u_{r} y_{r0} + w \hfill \\ St:\mathop \sum \limits_{i = 1}^{m} v_{i} x_{i0} = 1\quad \quad \mathop \sum \limits_{r = 1}^{s} u_{r} y_{rj} - \mathop \sum \limits_{i = 1}^{m} v_{i} x_{ij} + w \le 0 \hfill \\ u_{r} v_{i} \ge 0\quad (j = 1,2, \ldots n) \hfill \\ \end{gathered}$$

where *m*, *s*, and *n* are the number of inputs, the number of outputs, and the number of units, respectively. The existence of a free variable with a *w* signal is the difference of this relation with the constant returns to scale. Therefore, the variable *w* signal determines returns to scale for every unit. The type of returns to scale represents a decrease, the scale to scale is fixed, and the type of returns to scale increases if *w* < 0, w = 0, and w > 0, respectively [24]. Scale efficiency and management efficiency are obtained in this relation in addition to technical efficiency. Technical efficiency shows the level of the firm’s capability to maximize the production level concerning resources and production factors. Further, the scale efficiency of a unit is obtained from the ratio of the observed efficiency of that unit to the optimal efficient scale. Moreover, many variable returns (VRS) to scale technical efficiency studies are divided into “scale efficiency” and “net technical efficiency” (net technical efficiency is also called management efficiency). The mentioned efficiencies are between 0 and 1, and the closer to 1 and 0 indicate more and less efficiency, respectively [24]. Tobit regression was estimated to measure the effect of environmental factors on efficiency with Stata15. Hospital ownership, type of activity, the population covered by the hospital, and the size of the hospital are environmental factors. In this research, the ownership and the type of activity are determined as well. All hospitals have public ownership. Additionally, they are the subsets of the Ministry of Health and Medical Education, including educational and therapeutic activities. Thus, the hospital size and hospital populations were independent variables, and the obtained technical efficiency was a dependent variable. Therefore, the dependent variable for hospitals with more and less than 180 beds was calculated as one and zero according to the previous study, respectively [20]. Furthermore, hospitals with a covered population of more and less than 170,000 were scored as zero and one, respectively, according to the population dispersal in the west of Iran.

## Results

### Technical efficiency, management efficiency, and scale efficiency

According to technical efficiency, the results concerning the efficiency and rankings of the hospitals are presented in Table 1. Assuming variable returns to scale, the average technical efficiency, the average management efficiency, and the average scale efficiency of the hospitals were calculated as 0.935, 0.961, and 0.987, respectively.

**Table 1:** The ranking of the efficiency of public hospitals in the west of Iran by data envelopment analysis

Hospital ID	technical efficiency	management efficiency	Scale efficiency
1	1	1	1
2	1	1	1
3	1	1	1
4	1	1	1
5	1	1	1
6	1	1	1
7	1	1	1
8	1	1	1
9	1	1	1
10	0.964	1	0.964
11	0.955	1	0/0.955
12	0.897	0.891	0.994
13	0.862	0.885	0.962
14	0.848	0.838	1
15	0.554	0.661	0.823

**Fig. 1:**
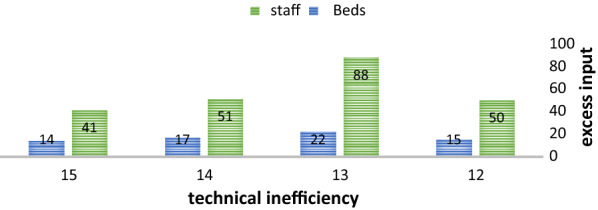
Excess inputs in inefficient hospitals

The calculated values by data envelopment analysis are presented as numbers 0–1. Further, Six hospitals with a lack of maximum technical efficiency. The minimum technical efficiency was 0.544, which was related to hospital No.15. Moreover, the scale efficiency of 60 and 40% of nine and six hospitals3 was 1 and less than 1, respectively. Furthermore, 73.3 and 26.7% of 11 and 4 hospitals had management efficiency of 1 and less than 1, respectively. In total, six and four hospitals were inefficient in technical efficiency and management efficiency, respectively. The excess of the related hospitals is specified by the number of active beds and staff based on management efficiency. Excess inputs are shown in Fig. 1.

According to the vertical axis representing the excess input, the most and least excess inputs of the number of staff belonged to hospitals No.13 and No.15, respectively. Further, the most and least excess inputs of the number of active beds were related to hospitals No.13 and No.15, respectively.

### Tobit regression as environmental factors

Hospital ownership, type of activity, the population covered by the hospital, and hospital size are not under management control. However, the public ownership of all hospitals and their educational and therapeutic activities were specified in this research. Thus, the size of the hospital and its population were considered as the environmental factors affecting efficiency. Moreover, Tobit regression was employed to assess their effect on efficiency(y*).

$${\text{y}}* = {\text{x}}\beta + {\text{u}},{\text{u}}|{\text{x}}\sim {\text{Normal}}\left( {0,\sigma {2}} \right)$$

But we only observe y = max(0, y*). The Tobit model uses MLE to estimate both β and σ for this model.

Furthermore, the T-test was applied to demonstrate the significance of coefficients. The results are provided in Table 2. According to the results of Tobit regression, hospital size has a positive and significant effect and population covered by the hospital has a negative and significant effect on hospitals' efficiency.

**Table 2:** The effect of environmental variables on the technical efficiency of hospitals with Tobit regression

Variable	Coefficients	SE	t
Hospital size	0.103	0.029	3.53
population covered by the hospital	− 0.092	0.027	− 3.34

## Discussion

According to the very significance of hospitals in providing health treatment services, as well as the health management system of every country, data envelopment analysis can be a huge step by providing the possibility for comparing, ranking, and patterning capability. Thus, it will pave the way for improving the functions of hospitals, particularly in the health treatment section. Based on the limitations of data envelopment analysis, the total considered organizations should be more than or equal to 3 times the inputs and outputs. Therefore, due to technical limitations, there is no possibility to choose more than five variables owing to the number of hospitals [24]. In the current study, the input-based and variable returns to scale method was applied since hospital outputs are not much in management control. This research assessed the efficiency of the hospitals and introduced inefficient units. Furthermore, the amount of the required resources in inefficient hospitals was estimated to achieve border efficiency. The results revealed that using the capacity of the existing hospitals and possible inputs can be a more usable solution compared to establishing new hospitals. The average technical efficiency, management efficiency, and scale efficiency of the considered hospitals were 0.935, 0.961, and 0.987, respectively. According to the results of data envelopment analysis and while assuming variable returns to scale, there was efficiency promotion without any increase in expenses. The findings of a study by Kiadliri [14] on technical efficiency assessment of the hospitals in the west of Iran showed that hospitals decrease their use of inputs, resulting in decreasing the number of expenses and wastes by improving the function and increasing efficiency in hospitals. The results further represented that 60% of hospitals have constant returns to scale. In other words, more than half of the hospitals operate at the most returns to the production scale, and the number of inputs and outputs is at the optimal level. Mehraban and Raghfar [25] reported similar results as well. Further, Mujasi [20] and Ali et al. [22] indicated the effect of environmental factors on efficiency using Tobit regression. On the other hand, the initial and optimal values in hospitals with technical efficiency less than 1 are different. The initial values must be improved to the optimal values to achieve optimal efficiency. Therefore, the number of staff excess inputs in hospitals No. 14, 13, 9, and 15 were 50, 88, 51, 41, and the number of excess inputs of the number of active beds was 15, 22, 17, 14, respectively.

Thus, hospitals must decrease their excess inputs to achieve maximum efficiency. Furthermore, the Tobit test results concerning environment variables in the studied method suggested that the size of the hospital and the population have a significant effect on efficiency. Therefore, the size of the hospital and the population of the location of the hospital will affect efficiency, which is out of management control.

## Conclusion

In general, six hospitals were inefficient in technical efficiency. Inefficiency was also observed in five and four hospitals in terms of scale efficiency and management efficiency, respectively. The size and population of the hospital as environmental factors have significant effects on hospital efficiency. Hospitals with the efficiency of less than one had different initial and optimal values, and there was also excess input. Therefore, the mentioned hospitals should decrease the initial values of their inputs to achieve maximum efficiency. According to the limitation of data envelopment analysis in this research, there was no possibility of choosing more than five variables due to the limited number of public hospitals in the west of Iran.

## Data Availability

The data that support the findings of this study are available from the corresponding author, [AF], upon reasonable request.

## Abbreviation

DEA: Data envelopment analysis
